# Indole-3-acetic acid treatment promotes postharvest kiwifruit softening by regulating starch and cell wall metabolism

**DOI:** 10.3389/fpls.2024.1485678

**Published:** 2024-11-12

**Authors:** Wenhao Guan, Mengze Cao, Wei Chen, Zhenfeng Yang, Xuewen Li, Li Wang, Liyu Shi

**Affiliations:** ^1^ Zhejiang Key Laboratory of Intelligent Food Logistic and Processing, College of Biological and Environmental Sciences, Zhejiang Wanli University, Ningbo, China; ^2^ Senior School, Seymour College, Glen Osmond, SA, Australia; ^3^ School of Food Science and Pharmacy, Xinjiang Agricultural University, Urumqi, China; ^4^ College of Tea and Food Sci Tech, Anhui Agricultural University, Hefei, China

**Keywords:** kiwifruit, IAA, softening, starch, cell wall

## Abstract

The softening process of postharvest kiwifruit is a critical aspect of fruit quality that has been extensively studied. However, the impact of indole-3-acetic acid (IAA) treatment on this process remains largely unexplored. In this study, we examined the effect of IAA treatment on the softening of postharvest kiwifruit. The results depicted that kiwifruit treated with IAA exhibited decreased firmness and increased ethylene production. Treatment with IAA upregulated the expression of starch decomposition genes, including *AcSEX* and *AcBAM*, resulting in a reduction in starch content. Additionally, IAA treatment induced cell wall breakdown, attributed to the enhanced transcript levels of cell wall-related degradation genes such as *AcPE*, *AcPG*, *AcPL*, and *AcCX* compared to the control. Consequently, IAA-treated kiwifruit displayed lower levels of cellulose and protopectin but higher levels of water-soluble pectin. In summary, our findings indicate that exogenous IAA promoted postharvest starch and cell wall biodegradation in kiwifruit, which reduced fruit firmness and accelerated fruit softening.

## Introduction

Originating from China, kiwifruit (*Actinidia chinensis* Planch) has gained popularity worldwide due to its unique flavor and rich nutritional profile. However, being a typical respiratory climacteric fruit, kiwifruit is usually harvested before physiological ripening, resulting in a firm texture and suboptimal taste. Furthermore, unlike other climacteric fruit such as bananas and mangoes, kiwifruit requires a longer duration to reach optimal ripeness for consumption (Lim et al., 2017). The post-ripening process is highly influenced by temperature and humidity, which severely affects consumer desire to purchase. Thus, softening firm kiwifruit to a palatable state before marketing plays a pivotal role in the development of the kiwifruit industry.

Fruit softening is a natural physiological process characterized by a decrease in firmness, indicative of the breakdown of starch and cell wall material (Liu et al., 2021; Wang et al., 2023). For fruit with high starch content, starch degradation plays a pivotal role in softening. This degradation process involves various enzymes, broadly categorized into phosphorylases and hydrolases. Starch phosphorylases mainly catalyze the phosphorylation reaction between the nonreducing ends of glucose residues and inorganic phosphoric acid in starch or glycogen molecules, and play an essential role in the metabolism of starch. Key enzymes in this group include phosphoglucan phosphatase (SEX), phosphoglucan water dikinase (PWD), glucan water dikinase (GWD) (Hu et al., 2016; Liu et al., 2023). For another, starch hydrolases facilitate the breakdown of starch into glucose. Examples of these enzymes include isoamylase (ISA), α-amylase (AMY), and β-amylase (BAM), but BAM is the key enzyme among these (Nascimento et al., 2006). Furthermore, enzymes involved in starch degradation encompass disproportionating enzyme (DPE), α-glucan phosphorylase (PHS), and limit dextrinase (LDA) (Dong et al., 2023).

Furthermore, modifications to cell wall composition significantly impact fruit firmness. The cell wall primarily comprises pectin, hemicellulose, and cellulose, and alterations in their composition contribute to fruit softening. As the fruit ripens, the activity of several hydrolytic enzymes associated with the cell wall increases, leading to notable changes in cell wall structure and polymer molecule size. For instance, cellulose chains shorten, hemicellulose polymers decrease in size, and especially pectin substances undergo substantial degradation (Ng et al., 2015; Villarreal et al., 2008). Research indicates the involvement of various enzymes in cell wall degradation, including pectinesterase (PE), polygalacturonase (PG), pectin lyase (PL), cellulase (CX), xyloglucan galactosyltransferase (XLT), xyloglucan endotransglycosylase/hydrolase (XTH), β-galacturonase Glycosidase (BGal), and xylanase (XYL) (Ali et al., 2022; Fullerton et al., 2020; Jhalegar et al., 2011). These enzymes catalyze the breakdown of pectin, hemicellulose, and cellulose, converting water-insoluble protopectin (PP) into water-soluble pectin (WSP), thereby resulting in decreased fruit firmness.

Currently, ethylene is the most commonly used treatment to promote softening of fruit and vegetables, widely employed in fruit such as blueberries (Wang et al., 2020), peaches (Cheng et al., 2022), apples (Wei et al., 2023). The effect of exogenous ethylene on the respiratory climacteric fruit is mainly to advance the respiratory peak, induce various physiological responses in the fruit, and accelerate the ripening and senescence process of the fruit (Bapat et al., 2010). Similarly, ethylene treatment has been applied to promote softening of kiwifruit fruit (Zhang et al., 2012). However, the strong ripening effect of ethylene can easily lead to over-ripening of fruit, which negatively impacts their edible quality and shelf life. In view of these issues with ethylene treatment, no suitable alternatives have been effectively implemented for softening kiwifruit. Nowadays, alternative phytohormones such as brassinosteroids (BR) and abscisic acid (ABA) have been used to promote fruit softening including persimmons and mango (He et al., 2018; Zaharah et al., 2013).

It has been reported that indole-3-acetic acid (IAA), a crucial auxin, not only regulates hormone levels within plants but also triggers resilience against external stresses (Jin et al., 2023), making it widely applicable in growth regulation and postharvest storage (Chen et al., 2016; Ma et al., 2021). Specifically, IAA treatment has been shown to boost the antioxidant capacity and enhance the quality of postharvest mango fruit (Zhou et al., 2023b). In addition, exogenous IAA can regulate the levels of endogenous hormones such as melatonin, abscisic acid, and γ-aminobutyric acid, thereby reducing chilling injury in peach fruit (Zhou et al., 2023a). Regarding kiwifruit, it has been reported that IAA treatment can activate defense mechanisms and fortify resistance against *Botrytis cinerea* (Li et al., 2023b). Since IAA is less effective at ripening fruit compared to ethylene, it serves as an excellent alternative for mitigating the issues of rapid deterioration in fruit edibility and shortened shelf life associated with ethylene. However, the role played by exogenous IAA in postharvest softening of kiwifruit remains unclear. Therefore, the objective of this research was to evaluate the influence of IAA on starch and cell wall content, thereby influencing softening in kiwifruit, and propose novel approaches to promote fruit softening. Additionally, transcript abundance of starch-degrading and cell wall-degrading genes were analyzed to elucidate the potential mechanism by which IAA treatment induced postharvest softening in kiwifruit.

## Materials and methods

### Plant material and treatments

‘Hongyang’ kiwifruit was harvested 120 d after pollination (soluble solids content 7.2% ± 0.3%, firmness around 42 N) from an orchard in Ningbo, Zhejiang Province, China. The harvested fruit were transported to the laboratory at room temperature, carefully chosen to ensure uniform size and freedom from any mechanical or human damage. Subsequently, they were divided into two groups, each consisting of ninety kiwifruits, with three biological replicates included in each group. The ideal IAA concentration, according to our pre-experiment data, is 0.5 mM. Consequently, the treatment group was immersed in a 0.5 mM IAA solution for 15 min, and the control group was treated with distilled water under the same conditions. Then, all fruit were dried naturally, bagged and stored at 20 °C and 80-90% relative humidity for eight days. Fruit samples were collected every two days during the storage period and fruit hardness and ethylene production were measured. Only the pulp was taken during fruit sampling, and the pulp was cut into cubes and immediately frozen with liquid nitrogen and stocked in a -80 °C freezer for subsequent determination of the indexes.

### Fruit firmness

Fruits were peeled on both longitudinal sides and their firmness was measured using a texture analyzer (TMS-Touch, US) with a 7.5 mm diameter probe, an inspection speed of 1 mm s^-1^, and a pulp deformation of 15%. Three fruits were measured in each biological replicate, and the result was expressed as N.

### Ethylene production

Three randomly selected fruit from each biological replicate were placed in 1.5 L gas collection containers and hermetically sealed at room temperature for 3 h. A syringe was used to draw 5 mL of gas from the top of each vessel. The gas was then passed through a rubber septum and fed into a gas chromatograph (GC-2014C, Shimadzu, Japan) equipped with a flame ionization detector (FID) to measure the released ethylene. The chromatographic process included the use of nitrogen as a carrier gas at a pressure of 68.6 kPa, a column flow rate of 2 mL min^-1^, a purge flow rate of 2 mL min^-1^, and a heating process in which the column temperature was initially set at 60 °C for 3.5 min, and then ramped up to 260 °C at a rate of 30 °C min^-1^. In addition, specific flow rates of N_2_ (64 mL min^-1^), H_2_ (40 mL min^-1^) and air (500 mL min^-1^) were used. The inlet temperature was maintained at 200 °C, and the detector temperature was set at 260 °C. The result was expressed as μL kg^-1^ h^-1^.

### Determination of starch content

Starch content was determined following the procedure of Stevenson et al. (2006) with slight modifications. The liquid nitrogen-frozen samples were ground into a powder, from which 0.05 g was taken and then mixed with 5 mL of 80% ethanol. After being centrifuged at 10,000 rpm for 15 min, the supernatant was discarded. The sample was then homogenized with 5 mL of ddH_2_O, followed by another round of centrifugation at 10,000 rpm for 15 min, after which the supernatant was once again discarded. Then the precipitate was dissolved in 2.5 mL of 80% calcium nitrate solution in a boiling water bath for 10 min, then centrifuged at 5000 rpm for 4 min, the supernatant was retained and the dissolution-boiling-centrifugation process was repeated three times. Finally, the solution was fixed to 10 mL with ddH_2_O. Following this, taken 2 mL of the extract and add 100 μL of iodine-potassium iodide buffer, and the absorbance was measured at 600 nm. The standard curve was drawn using 100 μg·mL^-1^ soluble starch standard solution, from which the starch content was obtained. The result was expressed as mg g^-1^.

### Determination of cell wall components

Water-soluble pectin and protopectin were quantified using a slightly modified method as described by Li et al. (2023a). Briefly, 0.1 g of ground frozen tissue was weighed and boiled in ethanol for 30 min, then cooled and centrifuged at 9,000 rpm for 12 min. Repeated the boiling-centrifugation process three times. Following this, ddH_2_O was added to the sediment to reach a constant volume of 20 mL, followed by heating at 50 °C for 30 min and centrifugation at 8000 rpm for 10 min. The supernatant was Water-soluble pectin. The remaining precipitate was boiled in 25 mL of 0.5 mol L^-1^ sulfuric acid for 1 h, then centrifuged for 10 min at 8000 rpm, and the resulting supernatant was identified as protopectin. The contents of protopectin and water-soluble pectin were determined by the same method, 0.25 mL of 1.5 g L^-1^ carbazole was added to 1 mL of pectin extract, and manually shaken until a white turbidity appeared. After the mixture is cooled, combined with 5 mL of concentrated sulfuric acid, and boiled for 20 min. After cooling, the absorbance at 530 nm was measured, and the content of the two pectins was calculated separately based on the standard curve of galacturonic acid. The cellulose (CEL) content was determined using the method described by Abu-Goukh and Bashir (2003). The result was expressed as mg g^-1^.

### Gene expression analysis by real-time quantitative PCR

Total RNA was extracted from kiwifruit tissues with the plant RNA kit (Omega Bio-Tek, USA). RNA was reversed to first-strand cDNA with RT SuperMix (Vazyme, Nanjing, China). The reverse transcription procedure was 50°C/15 min, 85°C/5 s. Samples were quantified on the StepOPlus™ system (BIO-RAD, USA) using gene-specific primers (**Supplementary Table S1**) and SYBR qPCR Master Mix (Vazyme, Nanjing, China). The PCR amplification profile was as follows: 95°C/3min (1 cycle), 95°C/10 s, 60°C/30 s (39 cycles). *AcActin* was used as an internal reference gene and data were analyzed by the 2^−ΔCt^ method.

### Statistical analysis

GraphPad Prism 9 software was used to analyze the experimental data. Multiple-sample t-tests were used to determine differences between the treatment and control groups (* p < 0.05, ** p < 0.01, and *** p < 0.001).

## Results

### Changes in firmness and ethylene production after IAA treatment

**Figure 1:**
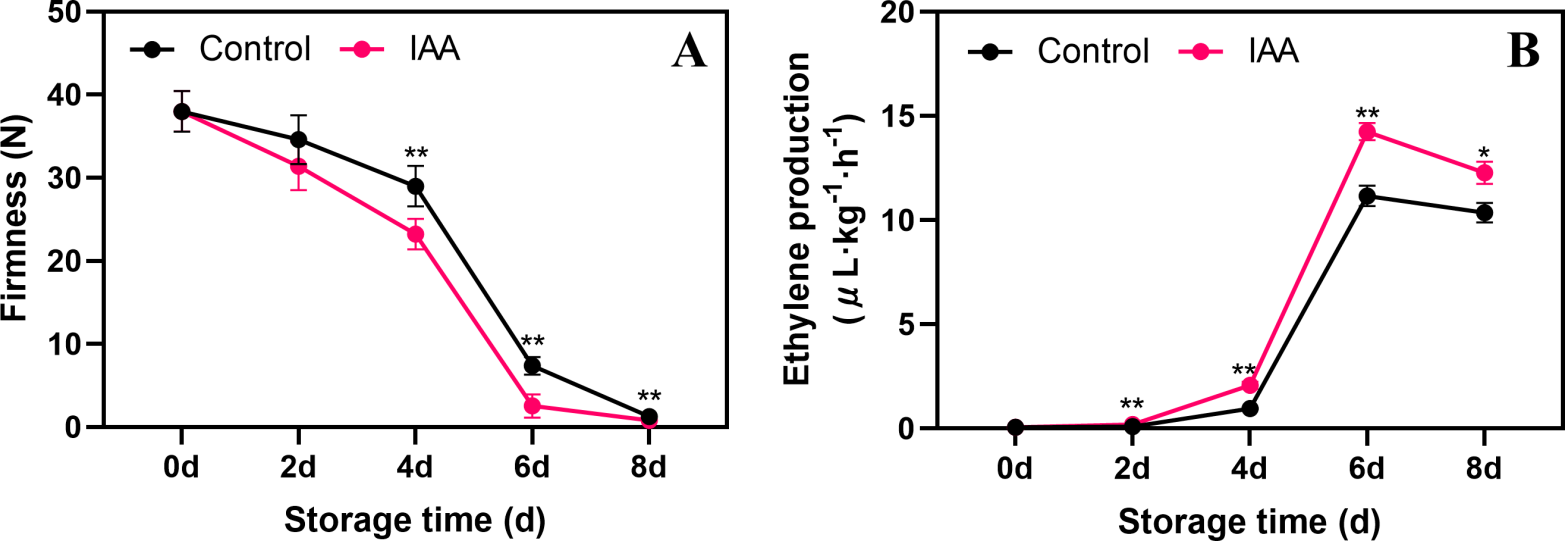
Effect of IAA treatment on fruit firmness **(A)** and ethylene production **(B)** of kiwifruit during storage at 20 °C. Vertical bars represent standard deviations of means. The asterisk indicates a statistical difference compared with the control (* p < 0.05 and ** p < 0.01).

The firmness of kiwifruit in both control and treated fruit gradually decreased throughout storage (**Figure 1A**). The IAA treatment significantly contributed to the decrease in fruit firmness starting on the 4th day of storage. During subsequent storage, the firmness was markedly lower in the treated group compared to the control group. IAA treatment noticeably increased ethylene production after 2 days of storage and peaked on day 6 (**Figure 1B**). Although ethylene production started to decrease by day 8, it remained higher in the treated group compared to the control.

### Changes in starch content after IAA treatment

**Figure 2:**
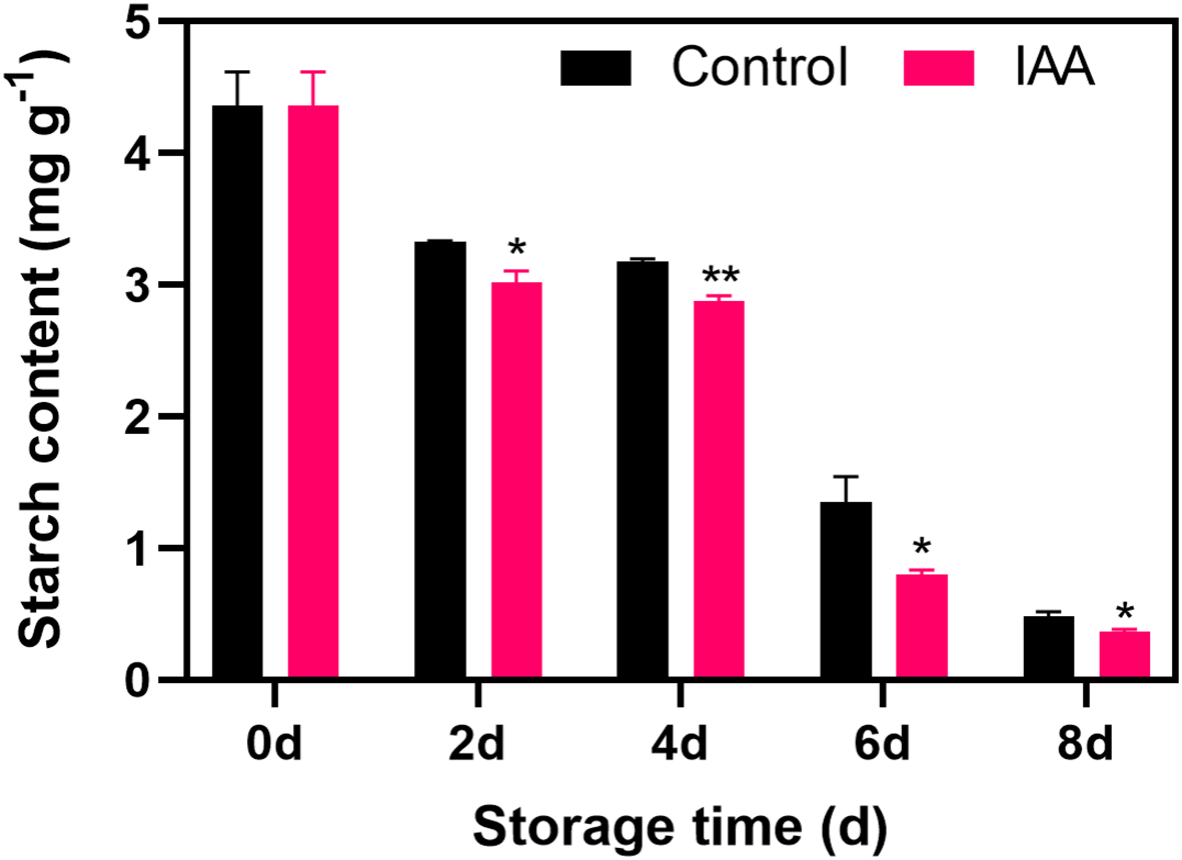
Effect of IAA treatment on starch content of kiwifruit during storage at 20 °C. Vertical bars represent standard deviations of means. The asterisk indicates a statistical difference compared with the control (* p < 0.05 and ** p < 0.01).

The starch content of IAA-treated and untreated kiwifruit showed a similar trend with a gradual decline within the storage (**Figure 2**). The treatment could induce the decrease in starch content and a significantly lower level was observed in IAA-treated fruit throughout the storage.

### Changes in starch degradation genes after IAA treatment

**Figure 3:**
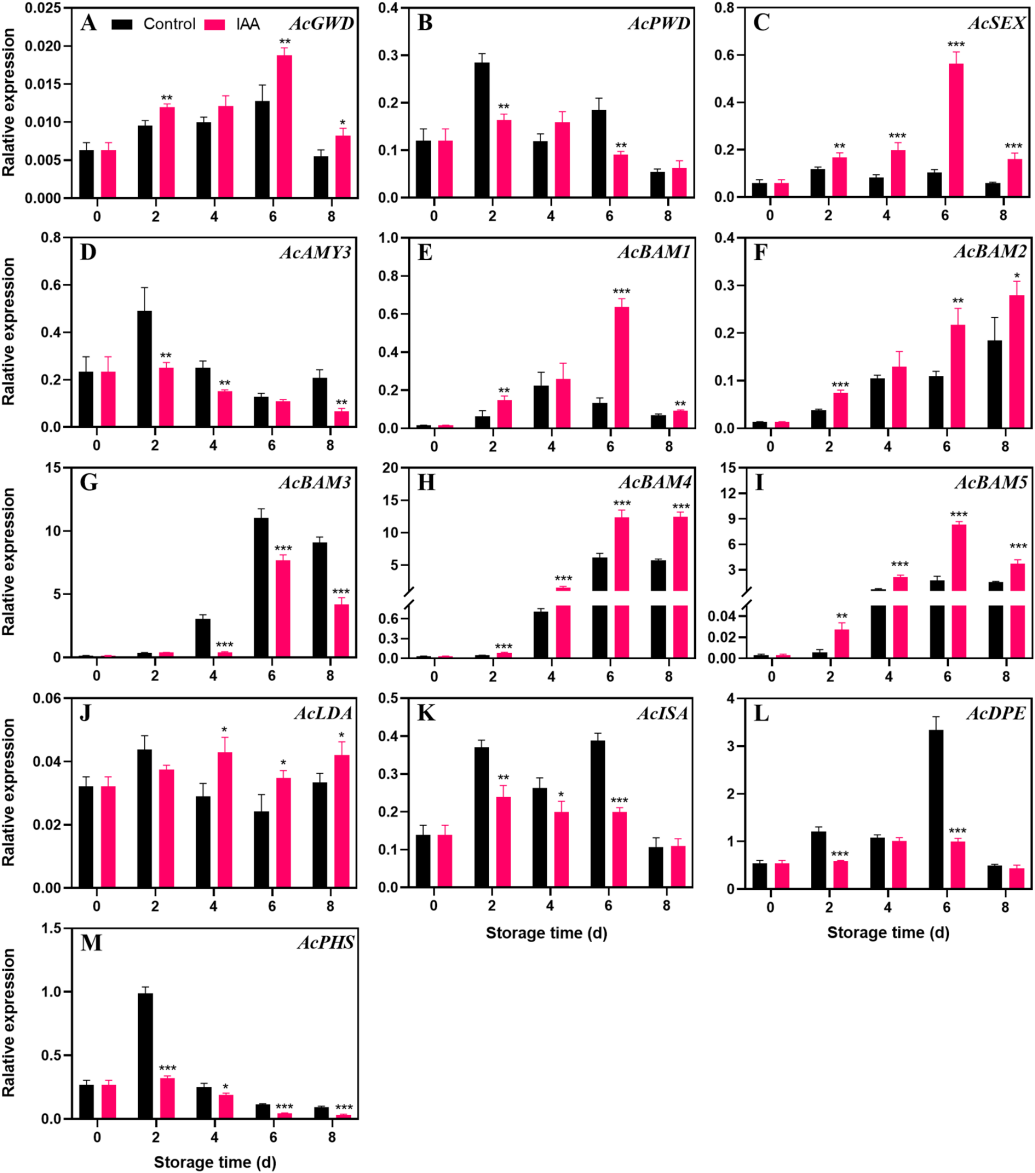
Effect of IAA treatment on starch degradation genes of kiwifruit during storage at 20 °C;. Expression levels of *AcGWD* **(A)**, *AcPWD* **(B)**, *AcSEX* **(C)**, *AcAMY3* **(D)**, *AcBAM1* **(E)**, *AcBAM2* **(F)**, *AcBAM3* **(G)**, *AcBAM4* **(H)**, *AcBAM5* **(I)**, *AcLDA* **(J)**, *AcISA* **(K)**, *AcDPE* **(L)**, and *AcPHS* **(M)** were analyzed. Vertical bars represent standard deviations of means. The asterisk indicates a statistical difference compared with the control (* p < 0.05, ** p < 0.01, and *** p < 0.001).

During storage, the expression level of *AcGWD* (**Figure 3A**) first increased and then decreased, in which the treatment significantly increased its expression. However, IAA treatment instead suppressed *AcPWD* (**Figure 3B**) expression. *AcSEX* (**Figure 3C**) reached the maximum expression level on day 6 in the treated fruit, which tripled, but remained at a low level in the control group. During the entire storage, the transcript levels of *AcSEX* in the treatment group were obviously increased compared to the control group. The expression of the gene *AcAMY3* (**Figure 3D**) reached the highest on the second day, and then gradually decreased. During this process, the treatment suppressed its expression. *AcBAM1* (**Figure 3E**) showed an increasing and then decreasing trend during storage, whereas the IAA treatment delayed the decrease and significantly increased the transcript abundance throughout storage. Transcript levels *AcBAM2* (**Figure 3F**) increased across storage, while treated kiwifruit had higher expression levels than the control. *AcBAM3* (**Figure 3G**) follows the same trend as *AcBAM1*, with the difference that IAA represses the transcript levels of *AcBAM3*. The expression levels of genes *AcBAM4* (**Figure 3H**) and *AcBAM5* (**Figure 3I**) were markedly higher than those of the control throughout the storage period, especially on days 6 and 8 when the expression levels increased significantly. At the later stage of storage, the expression level of *AcLDA* (**Figure 3J**) showed a significant increase compared to the control. IAA treatment did not promote the expression of *AcISA* (**Figure 3K**), *AcDPE* (**Figure 3L**), and *AcPHS* (**Figure 3M**) genes, and even had inhibitory effects.

### Changes in cell wall composition content after IAA treatment

**Figure 4:**
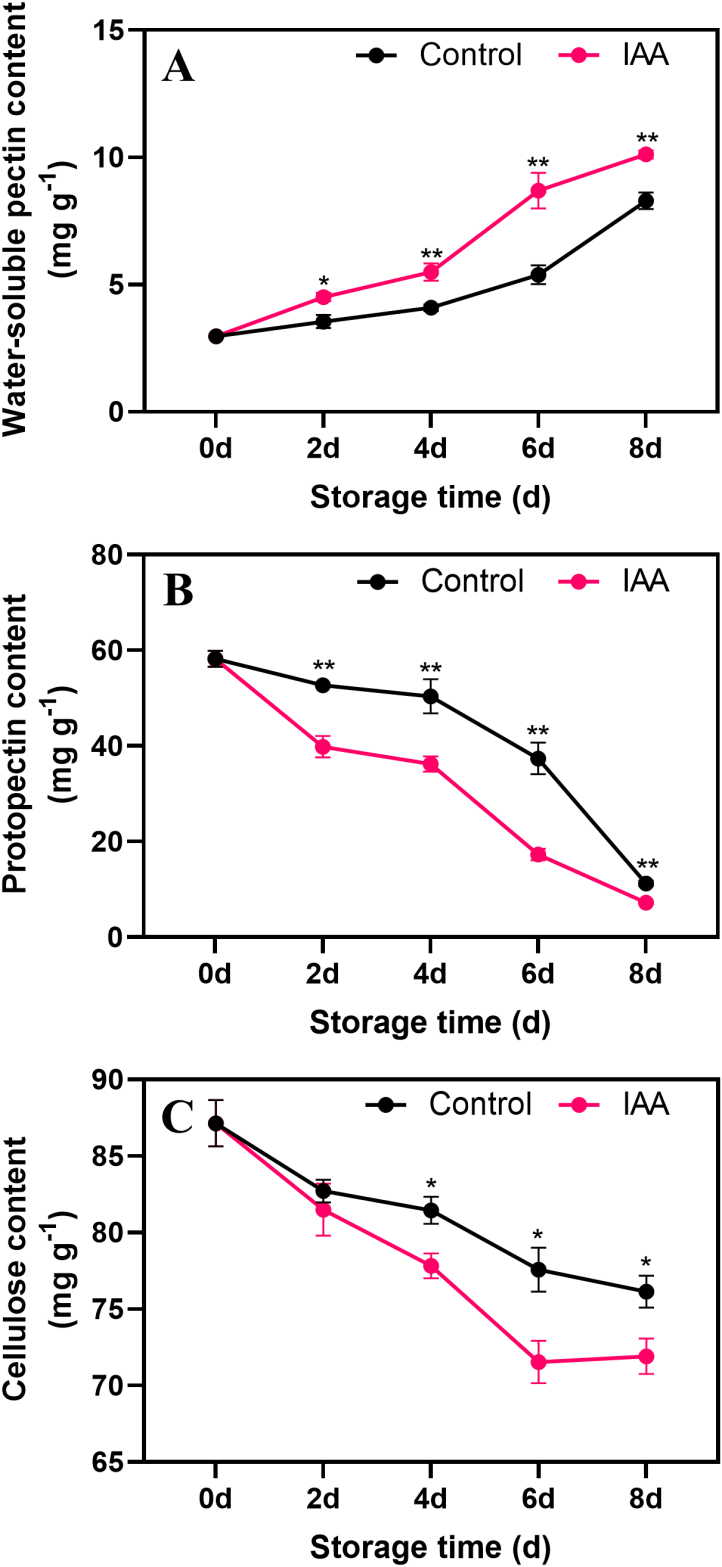
Effect of IAA treatment on water-soluble pectin **(A)**, protopectin **(B)** and cellulose **(C)** contents of kiwifruit during storage at 20 °C. Vertical bars represent standard deviations of means. The asterisk indicates a statistical difference compared with the control (* p < 0.05 and ** p < 0.01).

The content of WSP showed an increase during storage. Starting on the 2nd day of storage, water-soluble pectin in IAA-treated kiwifruit was higher than that in the control (**Figure 4A**). In contrast, the protopectin content continued to decline during storage. On days 2 to 8 of storage, the content of PP in IAA-treated kiwifruit fruit was significantly lower than that of the control (**Figure 4B**). Similar to protopectin, CEL content in control and treated fruit continued to decrease during storage, while CEL content in IAA-treated kiwifruit was lower than that of control on both days 6 and 8 (**Figure 4C**).

### Changes in genes for degradation of cell wall composition after IAA treatment

**Figure 5:**
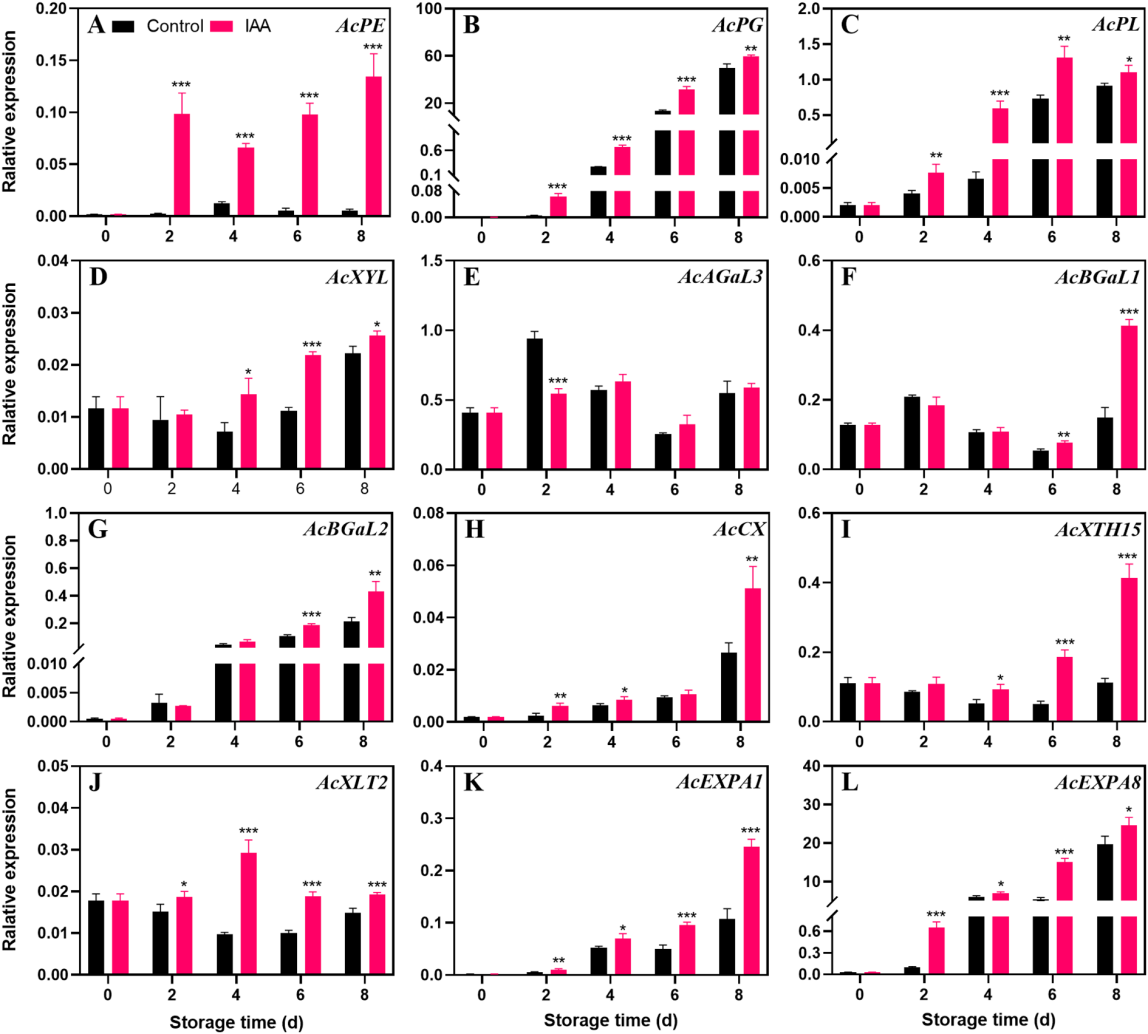
Effect of IAA treatment on cell wall degradation genes of kiwifruit during storage at 20 °C. Expression levels of *AcPE* **(A)**, *AcPG* **(B)**, *AcPL* **(C)**, *AcXYL* **(D)**, *AcAGaL3* **(E)**, *AcBGaL1* **(F)**, *AcBGaL*2 **(G)**, *AcCX* **(H)**, *AcXTH15* **(I)**, *AcXLT2* **(J)**, *AcEXPA1* **(K)**, and *AcEXPA8* **(L)** were analyzed. Vertical bars represent standard deviations of means. The asterisk indicates a statistical difference compared with the control (* p < 0.05, ** p < 0.01, and *** p < 0.001).

The transcription level of *AcPE* (**Figure 5A**) in the control group has always tended to be at a low level, while in the treatment group, the expression level has been increasing since the 2nd day. During the entire storage period, the transcript abundance in the treatment group was markedly higher than that in the control. *AcPG* (**Figure 5B**), *AcPL* (**Figure 5C**), and *AcXYL* (**Figure 5D**) genes showed a continuous upward trend in both treatment and the control groups, and IAA treatment markedly increased their expression during the entire storage period. IAA treatment did not promote the expression of the *AcAGaL3* (**Figure 5E**) gene in kiwifruit during storage, but significantly increased the expression level of the gene *AcBGaL1* (**Figure 5F**) on the 6th and 8th days of storage. For the genes *AcBGaL2* (**Figure 5G**), *AcCX* (**Figure 5H**), *AcEXPA1* (**Figure 5K**), and *AcEXPA8* (**Figure 5L**), their expression levels continued to increase throughout the storage, and the treatment group was notably higher than the control. The *AcXTH15* (**Figure 5I**) gene showed an upward trend in both treatment and the control groups. Notably, its expression level increased dramatically after the 6th day. The treatment group displayed significantly increased expression of *AcXTH15* during the entire storage. *AcXLT2* (**Figure 5J**) showed a downward trend in the control group, but in the treated fruit, it increased firstly and then decreased thereafter. The expression level of *AcXLT2* gene in the treated fruit was markedly higher than in the control during 4 to 8 days.

## Discussion

Firmness and ethylene production are the most obvious indicators of fruit softening and ripening. In light of this, research has pinpointed several exogenous treatments that significantly impact the fruit softening process. For example, acetylsalicylic acid can delay fresh-cut kiwifruit softening by maintaining firmness and reducing ethylene production (Wang et al., 2022), in contrast to abscisic acid, which promotes a decrease in firmness and accelerates softening in blueberries (Zhou et al., 2021). As a synthetic auxin analog, IAA is thought to have a stimulatory effect on plant growth (Tiwari et al., 2012). Meanwhile, it has been found that exogenous IAA increases ethylene production in peach fruit and promotes fruit softening (Liu, 2019). In addition, it has been observed that peach production of large amounts of system 2 ethylene is dependent on high concentrations of IAA (Tatsuki et al., 2013). Similarly, our study found that IAA treatment promoted a decrease in fruit firmness in kiwifruit (**Figure 1**), while accelerating ethylene production, suggesting IAA treatment promotes fruit softening in kiwifruit by enhancing ethylene production. Therefore, compared to directly using ethylene treatment, the ripening effect of IAA on the fruit is weakened, thus avoiding a rapid decline in edible quality and a shortened shelf life. Addition with the findings that that exogenous IAA treatment enhances kiwifruit resistance to *B. cinerea* (Li et al., 2023b), we can conclude that IAA treatment, as an exogenous regulator, promote post-harvest fruit ripening, maintain fruit quality, and safely and effectively control the occurrence of post-harvest fruit diseases.

As a crucial cellular component, starch in fruit plays a significant role in maintaining cell turgor and supporting fruit firmness. The process of starch degradation into soluble sugars marks the initiation of fruit softening. It has been shown that the decline in starch content primarily stems from enzymatic conversion, and is associated with alterations in intracellular metabolism and cell swelling pressure (Atkinson et al., 2011). In our investigation, IAA treatment resulted in a reduction in starch content (**Figure 2**), indicating that IAA treatment effectively expedited the conversion of starch to glucose, consequently diminishing fruit firmness.

Starch degradation commences with the hydrolysis of intact starch granules. Initially, the intact starch granule structure is disrupted by enzymes such as GWD and PWD, facilitating the action of starch hydrolase (Silver et al., 2014). Subsequently, the involvement of SEX is necessary to cleave phosphate groups from the starch, releasing maltose and oligosaccharides from the granules (Kotting et al., 2009). Finally, glucan is further broken down into glucose monomers by enzymes like AMY and BAM (Zeeman et al., 2010). In our present study, IAA promoted starch degradation in kiwifruit mainly by elevating the transcript levels of SEX and BAM (**Figure 3**), thereby affecting the activities of starch phosphatase and hydrolase. Similar observations have been reported in other studies, such as ethylene promoting kiwifruit softening by facilitating the expression of the BAM gene (Xu et al., 2024).

Alterations in cell wall composition are associated with the softening process of fruit. This process involves the disintegration of cell walls, reduction in intercellular aggregation, and weakening of the mechanical strength of the cytoskeleton, primarily driven by pectinolysis and cellulose hydrolysis. Pectin, a prominent polysaccharide found in plant cell walls and inner cell layers, contributes to enhancing intercellular adhesion and mechanical cell strength (Posé et al., 2019). Pectins are classified into water-soluble and water-insoluble forms based on their solubility. Protopectin, the water-insoluble pectin in plant cell walls, is chemically stable and abundant in early fruit ripening stages, gradually degrading into water-soluble pectin as fruit softens (Chea et al., 2019; Li et al., 2010). Studies have demonstrated that water-soluble pectin content increases during fruit softening, while protopectin and cellulose content decrease (Li et al., 2023a). In our investigation, IAA treatment elevated water-soluble pectin content and reduced protopectin levels, indicating its role in accelerating the conversion process from protopectin to water-soluble pectin, consequently promoting kiwifruit softening. Similar findings were reported in 1-MCP-treated loquats (Cao et al., 2009). Furthermore, cellulose breakdown disrupts cell wall structure and compromises its integrity, contributing to fruit softening. In our study, IAA-treated kiwifruit exhibited lower cellulose content compared to the control (**Figure 4**), indicating its significance in altering cell wall structure and promoting kiwifruit softening.

The breakdown of the cell wall is regulated by a variety of cell wall modifying enzymes (Vicente et al., 2007). PE catalyzes the deesterification of pectin, facilitating its subsequent hydrolysis by PG and PL (Micheli, 2001). Our study observed an increase in the expression levels of *AcPE*, *AcPG*, and *AcPL* genes in IAA-treated kiwifruit throughout the storage, suggesting that IAA promotes pectin degradation by upregulating degradation genes. Additionally, β-xyl, α-GaL, and β-GaL are crucial glycosidases involved in hydrolyzing sugar molecules on pectin branch chains (Wei et al., 2010). IAA treatment up-regulated the expression of *AcXYL*, *AcBGaL1*, and *AcBGaL2* genes, further facilitating pectin degradation. Similar findings were reported in studies on hydrogen sulfide-treated kiwifruit and low-voltage electrostatic field-treated strawberries (Lin et al., 2020; Xu et al., 2022). On the other hand, XTH, XLT and CX play crucial roles in cellulose and hemicellulose degradation (Ren et al., 2020; Xiang et al., 2023; Zhao et al., 2019). Our findings revealed that the transcript abundance of *AcCX*, *AcXTH15*, and *AcXLT2* genes was upregulated by IAA treatment, suggesting that IAA can influence cell wall composition by promoting cellulose degradation, thereby promoting kiwifruit softening. Similar to our study, electron beam irradiation has been shown to inhibit mango softening by regulating cellulase (Nguyen et al., 2021). Furthermore, expansin plays an auxiliary role in cell wall decomposition (Valenzuela-Riffo et al., 2020; Zhang et al., 2021). Overexpression of the expansin-like gene *MdEXLB1* has been found to accelerate fruit texture softening in tomatoes (Chen et al., 2022). Our study observed upregulation of the expression levels of *AcEXPA1* and *AcEXPA8* genes with IAA treatment, suggesting that IAA-mediated promotion of kiwifruit softening is also associated with genes encoding expansins. In conclusion, IAA treatment promoted the expression of most cell wall degradation genes during kiwifruit storage, accelerating the decomposition of pectin and cellulose, ultimately resulting in accelerated fruit softening (**Figure 5**).

Endogenous ethylene plays an essential role in fruit ripening by orchestrating various metabolic processes through coordination with multiple genes (Kumar et al., 2014). Studies have shown that salicylic acid inhibits apricot fruit softening by affecting ethylene biosynthesis, thereby reducing the transcript abundance of genes participating in cell wall disruption (Li et al., 2022). Similarly, in gibberellin-treated kiwifruit, endogenous ethylene levels were found to be closely associated with genes implicated in regulating starch and cell wall degeneration (Yang et al., 2023). Our research findings align with these observations, suggesting a potential correlation. Therefore, in addition to the direct effects of IAA on starch and cell wall degeneration, we hypothesize that IAA may indirectly influence starch and cell wall degeneration by promoting ethylene production.

## Conclusion

In conclusion, our results demonstrated that IAA induced a reduction in firmness and an elevation in ethylene production in postharvest kiwifruit. By upregulating the expression of relevant genes implicated in starch and cell wall degeneration, IAA treatment induced the increase in water-soluble pectin content while reducing the levels of starch, protopectin, and cellulose in kiwifruit. Therefore, it can be concluded that IAA enhanced kiwifruit softening by modulating starch and cell wall metabolism, either dependent or independent on ethylene production. Our study presented a novel approach to promote kiwifruit ripening after harvest. However, further investigations are required to elucidate the signaling pathways especially the cross-talk between ethylene and auxin through which IAA treatment regulates starch and cell wall degradation during kiwifruit softening.

## Data Availability

The original contributions presented in the study are included in the article/**Supplementary Material**. Further inquiries can be directed to the corresponding author.

## References

[B1] Abu-Goukh A.-B. A. Bashir H. A. (2003). Changes in pectic enzymes and cellulase activity during guava fruit ripening. Food Chem. 83, 213–218. doi: 10.1016/S0308-8146(03)00067-0

[B2] Ali S. Ullah M. A. Nawaz A. Naz S. Shah A. A. Gohari G.. (2022). Carboxymethyl cellulose coating regulates cell wall polysaccharides disassembly and delays ripening of harvested banana fruit. Postharvest Biol. Technol. 191, 111978. doi: 10.1016/j.postharvbio.2022.111978

[B3] Atkinson R. G. Gunaseelan K. Wang M. Y. Luo L. K. Wang T. C. Norling C. L.. (2011). Dissecting the role of climacteric ethylene in kiwifruit (*Actinidia chinensis*) ripening using a 1-aminocyclopropane-1-carboxylic acid oxidase knockdown line. J. Exp. Bot. 62, 3821–3835. doi: 10.1093/jxb/err063 21511911

[B4] Bapat V. A. Trivedi P. K. Ghosh A. Sane V. A. Ganapathi T. R. Nath P. (2010). Ripening of fleshy fruit: Molecular insight and the role of ethylene. Biotechnol. Adv. 28, 94–107. doi: 10.1016/j.bioteChadv.2009.10.002 19850118

[B5] Cao S. Zheng Y. Wang K. Rui H. Tang S. (2009). Effect of 1-methylcyclopropene treatment on chilling injury, fatty acid and cell wall polysaccharide composition in loquat fruit. J. Agric. Food Chem. 57, 8439–8443. doi: 10.1021/jf902114y 19711912

[B6] Chea S. Yu D. J. Park J. Oh H. D. Chung S. W. Lee H. J. (2019). Fruit softening correlates with enzymatic and compositional changes in fruit cell wall during ripening in 'Bluecrop' highbush blueberries. Scientia Hortic. 245, 163–170. doi: 10.1016/j.scienta.2018.10.019

[B7] Chen J. X. Mao L. C. Lu W. J. Ying T. J. Luo Z. S. (2016). Transcriptome profiling of postharvest strawberry fruit in response to exogenous auxin and abscisic acid. Planta 243, 183–197. doi: 10.1007/s00425-015-2402-5 26373937

[B8] Chen Y. H. Xie B. An X. H. Ma R. P. Zhao D. Y. Cheng C. G.. (2022). Overexpression of the apple expansin-like gene MdEXLB1 accelerates the softening of fruit texture in tomato. J. Integr. Agric. 21, 3578–3588. doi: 10.1016/j.jia.2022.08.030

[B9] Cheng C. X. Liu J. C. Wang X. K. Wang Y. Yuan Y. B. Yang S. L. (2022). PpERF/ABR1 functions as an activator to regulate PpPG expression resulting in fruit softening during storage in peach (*Prunus persica*). Postharvest Biol. Technol. 189, 111919. doi: 10.1016/j.postharvbio.2022.111919

[B10] Dong X. B. Chen L. K. Yang H. F. Tian L. H. Dong F. Q. Chai Y. R.. (2023). Pho1 cooperates with DPE1 to control short maltooligosaccharide mobilization during starch synthesis initiation in rice endosperm. Theor. Appl. Genet. 136, 47. doi: 10.1007/s00122-023-04250-z 36912930

[B11] Fullerton C. G. Prakash R. Ninan A. S. Atkinson R. G. Schaffer R. J. Hallett I. C.. (2020). Fruit from two kiwifruit genotypes with contrasting softening rates show differences in the xyloglucan and pectin domains of the cell wall. Front. Plant Sci. 11. doi: 10.3389/fpls.2020.00964 PMC7343912 32714354

[B12] He Y. H. Li J. Y. Ban Q. Y. Han S. K. Rao J. P. (2018). Role of Brassinosteroids in persimmon (*Diospyros kaki* L.) fruit ripening. J. Agric. Food. Chem. 66, 2637–2644. doi: 10.1021/acs.jafc.7b06117 29509414

[B13] Hu X. Kuang S. Zhang A. D. Zhang W. S. Chen M. J. Yin X. R.. (2016). Characterization of starch degradation related genes in postharvest kiwifruit. Int. J. Mol. Sci. 17, 2112. doi: 10.3390/ijms17122112 27983700 PMC5187912

[B14] Jhalegar M. J. Sharma R. R. Pal R. K. Arora A. Dahuja A. (2011). Analysis of physiological and biochemical changes in kiwifruit (*Actinidia deliciosa* cv. Allison) after the postharvest treatment with 1-Methylcyclopropene. J. Plant Biochem. Biotechnol. 20, 205–210. doi: 10.1007/s13562-011-0047-4

[B15] Jin M. R. Liu Y. L. Shi B. S. Yuan H. (2023). Exogenous IAA improves the seedling growth of *Syringa villosa* via regulating the endogenous hormones and enhancing the photosynthesis. Scientia Hortic. 308, 111585. doi: 10.1016/j.scienta.2022.111585

[B16] Kotting O. Santelia D. Edner C. Eicke S. Marthaler T. Gentry M. S.. (2009). STARCH-EXCESS4 is a laforin-like phosphoglucan phosphatase required for starch degradation in *Arabidopsis thaliana* . Plant Cell 21, 334–346. doi: 10.1105/tpc.108.064360 19141707 PMC2648081

[B17] Kumar R. Khurana A. Sharma A. K. (2014). Role of plant hormones and their interplay in development and ripening of fleshy fruits. J. Exp. Bot. 65, 4561–4575. doi: 10.1093/jxb/eru277 25028558

[B18] Li S. S. Qiu C. Y. Yang M. J. Shi L. Y. Cao S. F. Yang Z. F.. (2023a). Effect of gibberellic acid on cell wall degradation and softening in postharvest okras. LWT- Food Sci. Technol. 186, 115223. doi: 10.1016/j.lwt.2023.115223

[B19] Li X. Xu C. Korban S. S. Chen K. (2010). Regulatory mechanisms of textural changes in ripening fruits. Crit. Rev. Plant Sci. 29, 222–243. doi: 10.1080/07352689.2010.487776

[B20] Li Y. L. He H. Hou Y. Y. Kelimu A. Wu F. Zhao Y. T.. (2022). Salicylic acid treatment delays apricot (*Prunus Armeniaca* L.) fruit softening by inhibiting ethylene biosynthesis and cell wall degradation. Scientia Hortic. 300, 111061. doi: 10.1016/j.scienta.2022.111061

[B21] Li Z. X. Yang S. Wang X. Liao Q. H. Zhang W. L. Liu J.. (2023b). Widely targeted metabolomics analysis reveals the effect of exogenous auxin on postharvest resistance to *Botrytis cinerea* in kiwifruit (*Actinidia chinensis* L.). Postharvest Biol. Technol. 195, 112129. doi: 10.1016/j.postharvbio.2022.112129

[B22] Lim S. Lee J. G. Lee E. J. (2017). Comparison of fruit quality and GC-MS-based metabolite profiling of kiwifruit 'Jecy green': Natural and exogenous ethylene-induced ripening. Food Chem. 234, 81–92. doi: 10.1016/j.foodchem.2017.04.163 28551271

[B23] Lin X. C. Yang R. Dou Y. Zhang W. Du H. Y. Zhu L. Q.. (2020). Transcriptome analysis reveals delaying of the ripening and cell-wall degradation of kiwifruit by hydrogen sulfide. J. Sci. Food Agric. 100, 2280–2287. doi: 10.1002/jsfa.10260 31944323

[B24] Liu N. N. (2019). Effects of IAA and ABA on the immature peach fruit development process. Hortic. Plant J. 5, 145–154. doi: 10.1016/j.hpj.2019.01.005

[B25] Liu H. R. Liu L. H. Liang D. Y. Zhang M. Jia C. G. Qi M. F.. (2021). SlBES1 promotes tomato fruit softening through transcriptional inhibition of PMEU1. Iscience 24, 102926. doi: 10.1016/j.isci.2021.102926 34430815 PMC8374504

[B26] Liu J. Wang X. C. Guan Z. Y. Wu M. L. Wang X. Y. Fan R.. (2023). The LIKE SEX FOUR 1-malate dehydrogenase complex functions as a scaffold to recruit β-amylase to promote starch degradation. Plant Cell 36, 194–212. doi: 10.1093/plcell/koad259 37804098 PMC10734626

[B27] Ma G. Zhang L. C. Kudaka R. Inaba H. Murakami K. Yamamoto M.. (2021). Auxin induced carotenoid accumulation in GA and PDJ-treated citrus fruit after harvest. Postharvest Biol. Technol. 181, 61–74. doi: 10.1016/j.postharvbio.2021.111676

[B28] Micheli F. (2001). Pectin methylesterases: cell wall enzymes with important roles in plant physiology. Trends Plant Sci. 6, 414–419. doi: 10.1016/S1360-1385(01)02045-3 11544130

[B29] Nascimento J. Júnior A. V. Bassinello P. Z. Cordenunsi B. R. Mainardi J. A. Purgatto E.. (2006). Beta-amylase expression and starch degradation during banana ripening. Postharvest Biol. Technol. 40, 41–47. doi: 10.1016/j.postharvbio.2005.11.008

[B30] Ng J. K. T. Schröder R. Brummell D. A. Sutherland P. W. Hallett I. C. Smith B. G.. (2015). Lower cell wall pectin solubilisation and galactose loss during early fruit development in apple (*Malus × domestica*) cultivar 'Scifresh' are associated with slower softening rate. J. Plant Physiol. 176, 129–137. doi: 10.1016/j.jplph.2014.12.012 25602611

[B31] Nguyen T. T. Kato M. Ma G. Zhang L. C. Uthairatanakij A. Srilaong V.. (2021). Electron beam radiation delayed the disassembly of cell wall polysaccharides in harvested mangoes. Postharvest Biol. Technol. 178, 111544. doi: 10.1016/j.postharvbio.2021.111544

[B32] Posé S. Paniagua C. Matas A. J. Gunning A. P. Morris V. J. Quesada M. A.. (2019). A nanostructural view of the cell wall disassembly process during fruit ripening and postharvest storage by atomic force microscopy. Trends Food Sci. Technol. 87, 47–58. doi: 10.1016/j.tifs.2018.02.011

[B33] Ren Y. Y. Sun P. P. Wang X. X. Zhu Z. Y. (2020). Degradation of cell wall polysaccharides and change of related enzyme activities with fruit softening in *Annona squamosa* during storage. Postharvest Biol. Technol. 166, 111203. doi: 10.1016/j.postharvbio.2020.111203

[B34] Silver D. M. Kötting O. Moorhead G. B. G. (2014). Phosphoglucan phosphatase function sheds light on starch degradation. Trends Plant Sci. 19, 471–478. doi: 10.1016/j.tplants.2014.01.008 24534096

[B35] Stevenson D. G. Johnson S. R. Jane J. L. Inglett G. E. (2006). Chemical and physical properties of kiwifruit (*Actinidia deliciosa*) starch. Starch - Stärke 58, 323–329. doi: 10.1002/star.200600494

[B36] Tatsuki M. Nakajima N. Fujii H. Shimada T. Nakano M. Hayashi K.. (2013). Increased levels of IAA are required for system 2 ethylene synthesis causing fruit softening in peach (*Prunus persica* L. Batsch). J. Exp. Bot. 64, 1049–1059. doi: 10.1093/jxb/ers381 23364941 PMC3580816

[B37] Tiwari A. Offringa R. Heuvelink E. (2012). Auxin-induced fruit set in *Capsicum annuum* L. Requires downstream gibberellin biosynthesis. J. Plant Growth Regul. 31, 570–578. doi: 10.1007/s00344-012-9267-7

[B38] Valenzuela-Riffo F. Parra-Palma C. Ramos P. Morales-Quintana L. (2020). Molecular and structural insights into FaEXPA5, an alpha-expansin protein related with cell wall disassembly during ripening of strawberry fruit. Plant Physiol. Biochem. 154, 581–589. doi: 10.1016/j.plaphy.2020.06.010 32711363

[B39] Vicente A. R. Ortugno C. Rosli H. Powell A. L. Greve L. C. Labavitch J. M. (2007). Temporal sequence of cell wall disassembly events in developing fruits. 2. Analysis of blueberry (*Vaccinium species*). J. Agric. Food Chem. 55, 4125–4130. doi: 10.1021/jf063548j 17428068

[B40] Villarreal N. M. Rosli H. G. Martínez G. A. Civello P. M. (2008). Polygalacturonase activity and expression of related genes during ripening of strawberry cultivars with contrasting fruit firmness. Postharvest Biol. Technol. 47, 141–150. doi: 10.1016/j.postharvbio.2007.06.011

[B41] Wang Y. J. Li Y. X. Yang S. H. Li C. Li L. Gao S. Y.. (2023). Mechanism of ozone treatment in delayed softening of fresh-cut kiwifruit during storage. Postharvest Biol. Technol. 204, 112469. doi: 10.1016/j.postharvbio.2023.112469

[B42] Wang S. Y. Zhou Q. Zhou X. Zhang F. Ji S. J. (2020). Ethylene plays an important role in the softening and sucrose metabolism of blueberries postharvest. Food Chem. 310, 125965. doi: 10.1016/j.foodchem.2019.125965 31835222

[B43] Wang J. Zhu J. Z. Liu X. F. Allan A. C. Gao H. Y. Yin X. R.. (2022). Transcriptome analysis reveals mechanisms of acetylsalicylic acid-mediated fruit quality maintenance in fresh-cut kiwifruit. Postharvest Biol. Technol. 194, 112100. doi: 10.1016/j.postharvbio.2022.112100

[B44] Wei Y. Liu Z. Lv T. X. Xu Y. X. Wei Y. J. Liu W. T.. (2023). Ethylene enhances MdMAPK3-mediated phosphorylation of MdNAC72 to promote apple fruit softening. Plant Cell. 35, 2887–2909. doi: 10.1093/plcell/koad122 37132483 PMC10396387

[B45] Wei J. Ma F. Shi S. Qi X. Zhu X. Yuan J. (2010). Changes and postharvest regulation of activity and gene expression of enzymes related to cell wall degradation in ripening apple fruit. Postharvest Biol. Technol. 56, 147–154. doi: 10.1016/j.postharvbio.2009.12.003

[B46] Xiang M. Yuan S. Zhang Q. Liu X. H. Li Q. Y. Leng Z. M.. (2023). Galactosylation of xyloglucan is essential for the stabilization of the actin cytoskeleton and endomembrane system through the proper assembly of cell walls. J. Exp. Bot. 74, 5104–5123. doi: 10.1093/jxb/erad237 37386914

[B47] Xu C. Zhang X. Y. Liang J. Fu Y. J. Wang J. Jiang M.. (2022). Cell wall and reactive oxygen metabolism responses of strawberry fruit during storage to low voltage electrostatic field treatment. Postharvest Biol. Technol. 192, 112017. doi: 10.1016/j.postharvbio.2022.112017

[B48] Xu H. Zhu L. Lin Z. Wei W. Yang Y. Si J.. (2024). Banana MabZIP21 positively regulates MaBAM4, MaBAM7 and MaAMY3 expression to mediate starch degradation during postharvest ripening. Postharvest Biol. Technol. 211, 112835. doi: 10.1016/j.postharvbio.2024.112835

[B49] Yang H. Y. Li J. Z. Li X. H. Wu R. Zhang X. L. Fan X. G.. (2023). The mechanism of gibberellins treatment suppressing kiwifruit postharvest ripening processes by transcriptome analysis. Postharvest Biol. Technol. 198, 112223. doi: 10.1016/j.postharvbio.2022.112223

[B50] Zaharah S. S. Singh Z. Symons G. M. Reid J. B. (2013). Mode of action of abscisic acid in triggering ethylene biosynthesis and softening during ripening in mango fruit. Postharvest Biol. Technol. 75, 37–44. doi: 10.1016/j.postharvbio.2012.07.009

[B51] Zeeman S. C. Kossmann J. Smith A. M. (2010). Starch: its metabolism, evolution, and biotechnological modification in plants. Annu. Rev. Plant Biol. 61, 209–234. doi: 10.1146/annurev-arplant-042809-112301 20192737

[B52] Zhang L. H. Li S. F. Liu X. H. Song C. L. Liu X. (2012). Effects of ethephon on physicochemical and quality properties of kiwifruit during ripening. Postharvest Biol. Technol. 65, 69–75. doi: 10.1016/j.postharvbio.2011.11.004

[B53] Zhang P. Q. Su R. X. Duan Y. H. Cui M. Huang R. L. Qi W.. (2021). Synergy between endo/exo-glucanases and expansin enhances enzyme adsorption and cellulose conversion. Carbohydr. Polym. 253, 117287. doi: 10.1016/j.carbpol.2020.117287 33278952

[B54] Zhao Y. T. Zhu X. Hou Y. Y. Wang X. Y. Li X. H. (2019). Effects of nitric oxide fumigation treatment on retarding cell wall degradation and delaying softening of winter jujube (*Ziziphus jujuba* Mill. cv. Dongzao) fruit during storage. Postharvest Biol. Technol. 156, 110954. doi: 10.1016/j.postharvbio.2019.110954

[B55] Zhou Q. H. Bao Z. Y. Yu Y. Chen W. Yang Z. F. Cao S. F.. (2023a). IAA regulated levels of endogenous phytohormones in relation to chilling tolerance in cold-stored peaches after harvest. Postharvest Biol. Technol. 205, 112490. doi: 10.1016/j.postharvbio.2023.112490

[B56] Zhou Y. Huang L. Liu S. Y. Zhao M. Y. Liu J. M. Lin L. J.. (2023b). Physiological and transcriptomic analysis of IAA-induced antioxidant defense and cell wall metabolism in postharvest mango fruit. Food Res. Int. 174, 113504. doi: 10.1016/j.foodres.2023.113504 37986499

[B57] Zhou Q. Zhang F. Ji S. J. Dai H. Y. Zhou X. Wei B. D.. (2021). Abscisic acid accelerates postharvest blueberry fruit softening by promoting cell wall metabolism. Scientia Hortic. 288, 110325. doi: 10.1016/j.scienta.2021.110325

