# Supplementary material for: Indole-3-acetic acid treatment promotes postharvest kiwifruit softening by regulating starch and cell wall metabolism
Source: Front Plant Sci. 2024 Nov 12;15:1485678. doi: 10.3389/fpls.2024.1485678 (PMC11588445; doi:10.3389/fpls.2024.1485678)
Supplement: Supplementary file 1 [file Table1.docx]

**Table S1 Primers used for gene expression analysis**

| **Gene name** | **Accession number** | **Forward Primer** | **Reverse Primer** |
| --- | --- | --- | --- |
| *AcGWD* | Actinidia37409 | GGTTGAGGCTATTGGATA | GGCTGTTCATAGGATTTATTC |
| *AcPWD* | Actinidia35919 | TGTGGGATCAGTAATACC | GGCTGTTTCAATCTTTTCTA |
| *AcAMY3* | Actinidia31182 | GGTGGAATCTGAGGAAGTAG | TTCTGTTGGTGGTGGTAAC |
| *AcBAM1* | Actinidia25096 | TGGAAGAGATTGACAGAGAC | GAGGACTGGAAGAGTATCAC |
| *AcBAM2* | Actinidia31484 | TTGAAGTCAGTTAATGTTG | TATTGTTGTGGAGAATGT |
| *AcBAM3* | Actinidia10870 | CGGTGACAGAATCCTAGC | TGCCTCGTGTTGTAGTATC |
| *AcBAM4* | Actinidia03409 | CCACTCGACACCATATCA | CTACCAATCCCCACCAAG |
| *AcBAM5* | Actinidia35931 | ACTGGCACTACAAAACTA | TTCCCTGTCTCTCATTTC |
| *AcISA* | Actinidia10346 | TTGCCAGTGTTCTTTGTC | CTTCCCATTCCATTCAGC |
| *AcLDA* | Actinidia35775 | TGAATGGTGAGCCTGAGA | GGATAGGGTCGTGAAGAA |
| *AcDPE* | Actinidia37854 | CTCTTCTGGTTAGTGGTGTT | AAGCCTGCGAATCCTCTATAG |
| *AcPHS* | Actinidia38839 | GAAGGAAATTCCGGTTATG | GTGCTTAATATAGACATCTTTATC |
| *AcSEX* | Actinidia29748 | GCTTGTAACTCTTGCCAA | TCAGGGTAATTGCCCATC |
| *AcPL* | Actinidia19372 | GAATCAGTAGTCCAAGAAGTG | CGCCAACAATCGTCAATT |
| *AcPE* | Actinidia21838 | TGGGAGGAAGATGGAGGAGG | TTTCTCTGTACACACCCGCC |
| *AcPG* | Actinidia11185 | CCTGGCCACGGTATTAGCAT | CCTGAGACTTGATTGGGGCA |
| *AcXLT2* | Actinidia13527 | ACTGCGAGATGATGCTGAGG | GAAATCCCATGTGATGCGGC |
| *AcBGAL1* | Actinidia32144 | CGAGTCTCAGGGTGGTCCTA | GGTGAACCATCCAGTCCAGG |
| *AcBGAL2* | Actinidia19227 | GTAATTGCCGTCATCATA | CACTACCTCTTCAACTTG |
| *AcAGAL3* | Actinidia31616 | GTGTTGACGACCCTGCCTTA | AGGAGCCTTCATCAAAGCCC |
| *AcXTH15* | Actinidia11201 | ATGCTTGCGTACACTCTGCT | ACTTTTTCTGCACCCACCGA |
| *AcXYL* | Actinidia21530 | TGGAACAAGTGGGGTGGTTC | AATTCCCTACAGCACTGCCC |
| *AcCX* | Actinidia37451 | GGCTCACTTGGCCCCTTTAT | TGCACCCCTGAAATGGACTC |
| *AcEXPA1* | Actinidia31904 | CAGCCACAATTTCTGCCCAC | CCAGTTCCTCGACATGGCTT |
| *AcEXPA8* | Actinidia00998 | ATCTGGCACAATGGGAGGTG | CGGATCTCGTAGCAAGACCC |
| *AcActin* | Actinidia06662 | GCTTACAGAGGCACCACTCAACC | CCGGAATCCAGCACCAATACCAG |
